# Detection of a Fourth Orbivirus Non-Structural Protein

**DOI:** 10.1371/journal.pone.0025697

**Published:** 2011-10-12

**Authors:** Mourad Belhouchet, Fauziah Mohd Jaafar, Andrew E. Firth, Jonathan M. Grimes, Peter P. C. Mertens, Houssam Attoui

**Affiliations:** 1 Vector-Borne Viral Diseases Programme, Institute for Animal Health, Pirbright, United Kingdom; 2 Division of Virology, Department of Pathology, University of Cambridge, Cambridge, United Kingdom; 3 Division of Structural Biology, Henry Wellcome Building for Genomic Medicine, Oxford, United Kingdom; London School of Hygiene and Tropical Medicine, United Kingdom

## Abstract

The genus *Orbivirus* includes both insect and tick-borne viruses. The orbivirus genome, composed of 10 segments of dsRNA, encodes 7 structural proteins (VP1–VP7) and 3 non-structural proteins (NS1–NS3). An open reading frame (ORF) that spans almost the entire length of genome segment-9 (Seg-9) encodes VP6 (the viral helicase). However, bioinformatic analysis recently identified an overlapping ORF (ORFX) in Seg-9. We show that ORFX encodes a new non-structural protein, identified here as NS4. Western blotting and confocal fluorescence microscopy, using antibodies raised against recombinant NS4 from Bluetongue virus (BTV, which is insect-borne), or Great Island virus (GIV, which is tick-borne), demonstrate that these proteins are synthesised in BTV or GIV infected mammalian cells, respectively. BTV NS4 is also expressed in *Culicoides* insect cells. NS4 forms aggregates throughout the cytoplasm as well as in the nucleus, consistent with identification of nuclear localisation signals within the NS4 sequence. Bioinformatic analyses indicate that NS4 contains coiled-coils, is related to proteins that bind nucleic acids, or are associated with membranes and shows similarities to nucleolar protein UTP20 (a processome subunit). Recombinant NS4 of GIV protects dsRNA from degradation by endoribonucleases of the RNAse III family, indicating that it interacts with dsRNA. However, BTV NS4, which is only half the putative size of the GIV NS4, did not protect dsRNA from RNAse III cleavage. NS4 of both GIV and BTV protect DNA from degradation by DNAse. NS4 was found to associate with lipid droplets in cells infected with BTV or GIV or transfected with a plasmid expressing NS4.

## Introduction

The genus *Orbivirus* currently includes twenty two distinct virus species, with genomes composed of 10 segments of linear double stranded RNA (dsRNA), that are vectored by *Culicoides* midges, ticks, phlebotomine flies, anopheline or culicine mosquitoes. The three economically most important orbiviruses: *Bluetongue virus* (BTV) (the *Orbivirus* ‘type-species’) *African horse sickness virus* (AHSV) and *Epizootic hemorrhagic disease virus* (EHDV) are all transmitted by *Culicoides* biting-midges [1]. Several tick-borne orbiviruses can infect humans, including members of the *Changuinola virus, Corriparta virus, Lebombo virus, Orungo virus* and *Great island virus* (GIV) species.

The coding assignments of the 10 BTV genome segments were initially determined in 1983 [2], [3], [4]. Seven distinct structural proteins (VP1 to VP7) and 3 distinct non-structural proteins (NS1, NS2 and NS3) were identified in orbivirus infected cells, or after in vitro translation of viral RNA. In most cases each genome segment encodes a single protein from a single open reading frame (ORF), expect segment 9 (Seg-9) and segment 10 (Seg-10), both of which encode two nearly identical proteins initiated from in-phase AUG codons close together near the upstream termini (VP6 and VP6a encoded by Seg-9, and NS3 and NS3a encoded by Seg-10) [2], [5]. However, in vitro translation of BTV RNA segments reproducibly generated a number of smaller translation products of unknown significance, that were usually dismissed as unimportant by-products of translation [2].

The icosahedral orbivirus core-particle is constructed as two concentric protein shells, the sub-core layer which contain 120 copies/particle of the T2 protein (VP3 of BTV), and the core-surface layer composed of 780 copies/particle of the T13 protein (VP7 of BTV). VP1, VP4 and VP6 are minor enzymatic proteins that are packaged along with the ten genome segments within the central space of the virus core [6], [7]. The orbivirus outer-capsid layer is composed of two additional structural proteins (VP2 and VP5 of BTV), which mediate cell-attachment and penetration during initiation of infection. These outer-capsid proteins are more variable than the core proteins and most of the non-structural proteins, and the specificity of their reactions with neutralising antibodies determines the virus serotype (as exemplified by VP2 of BTV [8]). The relative number and locations of the BTV structural proteins have been determined in biochemical and structural studies using cryo-electron microscopy and X-ray crystallography [7], [9], [10], [11], [12].

NS1 is the most abundant protein in BTV infected cells, forming tubules that may be involved in translocation of progeny virus particles to the cell membrane [13], [14]. BTV NS2 can be phosphorylated by ubiquitous cellular kinases and is an important matrix protein of the granular viral inclusion bodies (VIB) that form within the cytoplasm of infected cells. VIB represent the primary site of virus replication and assembly. The smallest of the BTV non-structural proteins that were previously identified, are membrane glycoproteins NS3 and NS3a, which are expressed in large amounts in insect cells, but not in mammalian cells. They are involved in the release of progeny virus particles from infected cells [15]. In some orbiviruses (e.g. AHSV) NS3/NS3a are highly variable and it has been suggested that they may be involved in determination of both vector competence and virulence [16].

BTV-Seg-9 encodes the minor core protein VP6, which is a helicase. Recent bioinformatic analyses have identified a new overlapping ORF in Seg-9 of both insect-borne and tick-borne orbiviruses, although the putative protein (identified here as NS4) varies in size between 10 kDa and 22.5 kDa [17], [18].

We report the synthesis and detection of NS4, in the cytoplasm and nuclei of cells infected with insect-borne and tick-borne orbiviruses (represented by BTV and GIV respectively).

## Materials and Methods

### Ethics statement

All animal immunisation work was conducted according to the recommendations in the Animals (Scientific procedures) Act of the Home Office of the UK and the Directive on the protection of Animals used for Experimental and other scientific purposes of the EU. The protocol was approved by the Ethics Committee of animal experiments at the Institute for Animal Health in the UK (Project license number 70/7060). All surgery was performed under sodium pentobarbital anaesthesia, and all efforts were made to minimize suffering.

### Cell cultures and viruses

BHK-21 (American type cell culture collection) were grown at 37°C under 5% CO2 in Glasgow's minimum essential medium (GMEM), supplemented with 10% foetal bovine serum, 10% tryptose phosphate broth, penicillin G (100 IU/ml) and streptomycin (100 µg/ml). *Culicoides sonorensis* KC cells were grown at 28°C in Schneider's insect medium supplemented with 15% fetal bovine serum.

Confluent monolayers of BHK-21 cells were infected with either BTV-8 (isolate NET2006/04) or Great Island virus (GIV) (isolate CAN1971/01) at a multiplicity of infection (MOI) of 0.1 pfu/cell. Infected cell cultures were incubated at 37°C for 72 hours until cell lysis began. The cells were then scraped into the supernatant and centrifuged at 3,000 g for 10 minutes. RNA was extracted from cell pellets using guanidinium isothiocyanate (RNA NOW reagent: Biogentex, Tx, USA) as described earlier [19].

KC cells were infected at an MOI of 0.1 pfu/cell and then incubated at 28°C for 7 days. Both BHK-21 and KC cell pellets were used in western blot analyses as described below.

Viruses were purified from BHK-21 infected cells, as previously described using a discontinuous sucrose gradient [20]. Virus particles formed a blue opalescent band at the interface of the sucrose solutions. This was recovered and further purified by layering onto a continuous Percoll® gradient as previously described [21], using an SW41 rotor (100000 g, 1 hour, 4°C). The virus formed a blue band which was collected, diluted in 0.1 M Tris-HCl and pelleted at 10000 g for 1 hour.

### Bioinformatic analyses of the overlapping ORF in Seg-9 of BTV and GIV

The hydrophobicity profile of different NS4 proteins was analysed using the Kyte and Doolittle hydrophobicity plot with a window size of 11 amino acids (aa) [22]. Sequence relatedness to proteins in public databases was assessed using the NCBI's BLAST (http://blast.ncbi.nlm.nih.gov/Blast.cgi)) and the pfam software (http://pfam.sanger.ac.uk/search/sequence). Amino acid alignments of NS4 of various orbiviruses were generated using the Clustal X program [23] and pairwise aa identities calculated using the MEGA 4 package [24]. The presence of ‘coiled-coils’ was indicated by analyses using the program ‘COILS’ (http://www.ch.embnet.org/cgi-bin/COILS_form_parser) and the PredictProtein server (http://www.predictprotein.org). The presence of nuclear localisation signals were analysed by PredictNSL, implemented in the PredictProtein server, and the cNLS Mapper (http://nls-mapper.iab.keio.ac.jp/cgi-bin/NLS_Mapper_form.cgi). Synonymous site conservation within the BTV VP6 coding sequence was analysed as described previously [25]. For this procedure, alignment columns in which the reference sequence (GenBank accession number: NC_006008) contained gap characters were removed so that the plots are in reference sequence coordinates.

### Cloning of BTV and GIV NS4

The RNA of BTV-8 or GIV was separated by 1% agarose gel electrophoresis. Seg-9 was cut from the gel using a clean scalpel blade, purified using RNaid kit (MP Biomedicals) and cDNA was synthesised using a single primer amplification technique as previously described [19]). The ORFs in Seg-9 from BTV-8 (between nucleotides 182 and 415, accession number: AM498059) and GIV (between nucleotides 176 and 748: accession number HM543473) were PCR amplified using specific primers tailed with restriction enzyme sites shown in table 1.

**Table 1:** Primer sequences used for cloning of NS4 ORF into pGEX-4T-2 or pCI-neo.

Primer	Sequence (5′→3′)	Plasmid	Segment	ORF position	Orientation
**NS4-BTVfor**	tacg**GAATTCacc** ATGGTGAGGGGGCGCAGTCG	pGEX-4T-2/pCI-Neo	9 BTV	182-201	Forward
**NS4-BTVrev**	tgag**GCGGCCGC** *TCACTA* TACCCATCTTCCTCCATTC	pGEX-4T-2/pCI-Neo	9 BTV	412-396	Reverse
**NS4-GIVfor**	atcg**GAATTCacc** ATGAGTTACCGGCAGGAGCA	pGEX-4T-2/pCI-Neo	9 GIV	176-195	Forward
**NS4-GIVrev-pGEX**	tgat**CTCGAG** *TCACTA* TTGCTGAACGCACCTTGTCC	pGEX-4T-2	9 GIV	748-726	Reverse
**NS4-GIVrev-pCI**	tgat**TCTAGA** *TCACTA* TTGCTGAACGCACCTTGTCC	pCI-neo	9GIV	748-726	Reverse

Underlined sequences are specific to the NS4 ORF. Sequences in italics in the reverse primers indicate two successive stop codons. Sequences in bold characters are restriction enzyme sites (in NS4-BTVfor and NS4-GIVfor, the site is EcoRI; in NS4-BTVrev the site is NotI, in NS4-GIVrev-pGEX the site is XhoI and in NS4-GIVrev-pCI the site is XbaI). Sequences in lower case characters are non-specific nucleotides added for an efficient restriction enzyme digestion.

The pGEX-4T-2 vector and Seg-9 PCR products were double-digested with EcoRI and NotI (BTV-8) or EcoRI and XhoI (GIV) enzymes (Invitrogen). The pCI-neo vector and Seg-9 PCR products were double-digested with EcoRI and NotI (BTV-8) or EcoRI and XbaI (GIV) enzymes. Digested products were gel purified using Genclean kit (Qbiogen). Corresponding vectors and PCR products were ligated overnight (O/N) at 16°C using T4 DNA ligase (Roche) to generate pGEX-BTVNS4, pGEX-GIVNS4, pCI-BTVNS4 or pCI-GIVNS4. These recombinant plasmids were used to transform XL1-Blue bacteria (Stratagene). Clones were recovered and grown in trypticase-soy-casein (TSC) medium containing 100 µg/ml ampicillin. The plamsids were subsequently purified using Qiaquick plasmid miniprep kit (Qiagen) and sequenced using the D-Rhodamine DNA sequencing kit and an ABI prism 377 sequence analyser (Perkin Elmer).

### Expression of BTV and GIV NS4 in bacteria

Confirmed pGEX-BTVNS4 or pGEX-GIVNS4 plasmids were used to transform BL21 or C41 bacteria. A single colony of each plasmid was grown overnight (ON) in TSC/ampicillin, then used to seed 200 ml of fresh TSC/ampicillin. The bacteria were grown until OD600 0.5, then 0.5 mM IPTG was added for induction, for 4 hours at 37°C, or for 8 hours at 28°C. The bacterial cells were pelleted and processed using Bugbuster protein purification (Novagen) as previously described [26]. The soluble fraction of the fusion protein was purified by glutathione affinity chromatography using glutathione sepharose, as directed by the manufacturer (GE Healthcare). Proteins were analysed by sodium dodecyl sulfate/polyacrylamide gel electrophoresis (SDS-PAGE) using a 10% polyacrylamide separating gel (Miniprotean III) with a 3% stacking gel and stained with Coomassie brilliant blue, as described previously [21]. The purified fusion protein was used to immunize rabbits (Harlan) with an initial injection, followed by 4 boosts at 2 weeks interval in the presence of Montanide ISA50 (Seppic) as an adjuvant.

### Western blot analysis of purified BTV and GIV virus particles and infected cell cultures

BTV-8 or GIV infected BHK-21 cells (5×10^6^ cells) and BTV-8 infected KC cells (5×10^6^ cells) were dissolved for 10 min at 100°C in 1 ml of sample denaturation buffer (160 mM Tris-HCl, 4 mM EDTA, 3.6% SDS, 60 mM DTT, 0.2% ß-mercaptoethanol, 0.8% methionine, 800 mM sucrose). A volume of 20 µl was analysed per well, by electrophoresis in a minigel (Miniprotean III tank - Bio-Rad). Purified and pelleted virus particles were also dissolved in sample buffer and analysed by SDS-PAGE, using a 4-20% gradient polyacrylamide gel.

Resolved proteins were electro-blotted on 0.2 µm nitrocellulose membrane (Bio-Rad) using 20 mM Tris, 0.05% SDS, 150 mM glycine and 20% V/V isopropanol transfer buffer. Membranes were blocked with 5% skimmed milk, in Tris buffered saline (TBS: 25 mM Tris/HCl, 150 mM NaCl, 2 mM KCl, pH 7.4) and incubated over night with a dilution of 1/300 rabbit antisera. Membranes were washed three times with TBS-Tween-20 (TBS containing 0.05% Tween-20) and further incubated with monoclonal, anti-rabbit, peroxydase conjugate (Sigma), diluted at 1/750 in 5% skimmed milk. After 2 hours the membrane was washed three times with TBS-Tween-20 and developed using 4-chloro-naphthol (Sigma) in presence of hydrogen peroxide.

### Preparation of the nuclear fraction of BHK-21 cells infected with BTV-8

Logarithmically growing BHK-21 cells were infected with BTV-8 (1pfu/cell) for 24 hours then harvested and washed once with PBS. Nuclear extracts were prepared from 2.5×10^7^ cells using the NE-PER nuclear and cytoplasmic extraction reagent kit (Pierce), as directed by the manufacturer. The nuclear extract was mixed volume to volume with sample denaturation buffer and analysed by SDS-PAGE using a 4–20% gradient polyacrylamide gel. Resolved proteins were electro-blotted on 0.2 µm nitrocellulose membrane as described above, blocked with 5% skimmed milk, in TBS-Tween-20 and incubated over night with a dilution of 1/300 anti-BTV-8 NS4 rabbit antiserum. The membranes were washed three times with TBS-Tween-20 and further incubated with monoclonal, anti-rabbit, peroxydase conjugate (Sigma), diluted at 1/750 in 5% skimmed milk. After 2 hours the membranes were washed three times with TBS-Tween-20 then incubated with Lumilight plus (Roche) chemiluminescent detection reagent, as described by the manufacturer. X-Omat radiographic films (Kodak) were exposed for 10 minutes to membranes then developed as described by the manufacturer.

### Localization of NS4 in infected cells by confocal fluorescence microscopy

BHK-21 cells were grown on coverslips placed at the bottom of a 24 well plates. 50% confluent cells were infected with 0.1 pfu/cell of BTV-8 or GIV, incubated at 37°C for 4 hours or 24 to 72 hours, then fixed in 4% paraformaldehyde and processed for immuno-fluorescence. Briefly, rabbit antisera raised against NS4 of BTV-8 or GIV and a mouse anti-alpha tubulin antibody were both diluted 1/500 in PBS containing 0.5% bovine serum albumin (PBS-A) and applied to the fixed cell. After 1 hour incubation at room temperature (RT), slides were washed in PBS, then incubated with Alexa Fluor 488 conjugated anti-rabbit IgG (Invitrogen) and Alexa Fluor 568 conjugated anti-mouse, both diluted 1/250 in PBS. After labelling with primary and secondary antibodies, the cells were stained with DAPI (1∶10,000) for 15 minutes for nuclear staining and mounted with Vectashield (Vector Laboratories) for confocal microscopy.

### Localization of NS4 by confocal fluorescence microscopy in cells transfected with pCI-BTVNS4 or pCI-GIVNS4

BHK-21 cells grown in 24 well plates (75% confluence), were transfected in triplicate, with pCI-BTVNS4 or pCI-GIVNS4 (4 µg/well) using Fugene-6 (Roche). At 48 hours post-transfection, the cells were fixed in 4% paraformaldehyde and processed for immuno-fluorescence, using anti-NS4 antibodies, as described above.

### Identification of NS4 in cells transfected with pCI-BTVNS4, using anti-BTV-8 immune serum from infected mice

BHK-21 cells were transfected with pCI-BTVNS4 using Fugene-6. At 48 hours post-transfection, the cells were dissolved in sample denaturation buffer as described above. Cell lysates were analysed by SDS-PAGE/Western blot, using an immune serum (diluted 1/50, in 5% skimmed milk) from mice infected with BTV-8. Non-transfected cells were used as control.

### Nucleic acid protection assays

dsRNA binding proteins can compete with Dicer (an endoribonuclease of the RNAse III family), reducing its ability to cleave long dsRNAs into 21 bp-long ‘interfering’ RNAs [27]. A dsRNA ladder (New England Biolabs) with sizes ranging from 500 to 21 bp was used as a template for Dicer cleavage. A competition assay with Dicer (Mobitech), was carried in the presence of 150 ng of expressed BTV-8 or GIV NS4, in a final volume of 20 µl containing 8 µl of the Dicer reaction, 2 µg of dsRNA, 1 mM ATP, 2.5 mM MgCl2. The reaction was incubated at room temperature for 20 minutes, followed by addition of a 1.5 U of Dicer, then incubated for a further 6 hours at 37°C. The reaction products were analysed by 3% agarose gel electrophoresis.

DNAse I is an endodeoxyribonuclease that can degrade dsDNA into 5′ phosphorylated tetranucleotides [28]. A dsDNA ladder (Promega) with sizes ranging from 2645 to 36 bp provides a target for DNAse I cleavage. Competition assays between DNAse I (Roche) and BTV-8 or GIV NS4, were carried out in a final volume of 20 µl, containing 2 µl of 10X DNAse I buffer and 2 µg of dsDNA. The reaction was incubated at room temperature for 20 minutes, followed by addition of a 2 U of DNAse I, then incubated for a further 30 minutes at 37°C. The completed reaction was heated at 99°C for 1 minute to inactivate the DNase and the reaction products were analysed by 2% agarose gel electrophoresis.

The outer capsid protein VP9 of Banna virus (BAV, genus *Sedaornavirus*, family *Reoviridae*) expressed in *E.coli* [29] was used as a control in both RNAse and DNAse assays.

### Interaction of NS4 with dsRNA

A colorimetric assay was developed to detect interactions between NS4 and dsRNA. Synthetic dsRNA was prepared with the 5′-end of one strand linked to biotin via a 15-atom mixed polarity tetraethylene glycol spacer (5′-Biotine TEG). This design allows the dsRNA to be captured at the bottom of a well of 96 well plate coated with streptavidin, while keeping the dsRNA free as a target for NS4 binding. The sequence of the +ve strand is: 5′-Biotine-TEG-UGGAAGCGGCUGGCAAUUAAUUUUGGUGUC-3′ and that of the negative strand is 5′-GACACCAAAAUUAAUUGCCAGCCGCUUCCA-3′. Increasing concentrations (from 1 to 640 ng) of the dsRNA in PBS were added to separate wells of a streptavidin-coated 96 well plate (Pierce) and allowed to bind at room temperature for 2 hours. The wells were washed three times with TBS-Tween-20, then two hundred microlitres of a 5% solution of bovine serum albumin (BSA) in PBS, was added in each well, to block non-specific sites. After washing 3 times with TBS-Tween-20 a fixed amount (150 ng) of either BTV or GIV NS4 in binding buffer (20 mM Tris-HCl pH 7.5, 50 mM KCl, 2 mM MgCl2, 2 mM MnCl2 and 5% glycerol) was added per well, prior to incubation for 30 minutes at 25°C. After the wells had been washed 3 times with TBS-Tween-20, rabbit anti-BTV or anti-GIV NS4 sera was diluted 1/250 in 5% BSA and 100 µl was added to each well, then the plates were incubated at 25°C for 2 hours. After washing three times, 100 µl of peroxydase conjugated anti-rabbit antibody was added (diluted 1/750 in 5% BSA) to each well. The plates were incubated at 25°C for 2 hours, then washed 3 times with TBS-Tween-20. One hundred microliters of SureBlue TMB 1-component microwell peroxidase substrate (tetramethyl benzidine from KPL) was added per well, then incubated for 30 minutes at 25°C. The reaction was stopped by adding 100 µl of 1 M HCl and the plate was read at OD 450 nm.

Wells not containing dsRNA/NS4, were included as negative controls. Wells from which the dsRNA was omitted, but in which NS4 (BTV or GIV) alone was incubated were also included as controls.

## Results

### Bioinformatic analyses

The program MLOGD models and compares sequence evolution in single-coding and dual-coding sequences. It has previously been used to identify a second ORF, in a different but overlapping reading frame from that encoding the viral helicase (VP6 of BTV), within Seg-9 of the insect-borne orbiviruses [17], [18], [30]. This ORF was also identified in tick-borne orbiviruses [18]. The length of the putative translation product is highly variable, even between closely related *Orbivirus* species. In BTV and EHDV it is approximately 10 kDa, in Peruvian horsesickness virus (PHSV) and Yunnan orbivirus (YUOV) it is approximately 13.5 kDa, while in AHSV it is approximately 17 kDa, and in GIV it is approximately 22.5 kDa (twice as long as in BTV). These NS4 sequences contain a high proportion of charged residues, with basic R+K (arginine + lysine) content ranging from 13% to 22%, while acidic E+D (glutamic + aspartic acids) content ranges from 12% to 22%. Each NS4 protein contains 4–5 histidine residues, with the exception of the BTV protein, which contains none.

**Figure 1:**
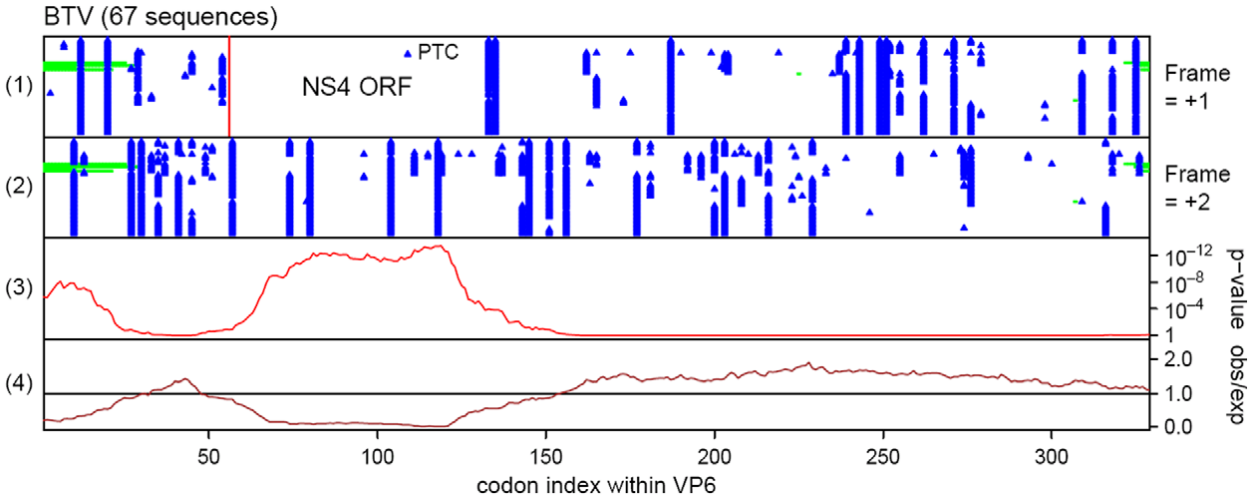
Synonymous site conservation in BTV VP6 coding sequence. Comparison of 67 BTV Seg-9 sequences. Panels 1 and 2 show the positions in the aligned sequences, of stop codons (blue triangles) in the +1 and +2 reading frames relative to the VP6 reading frame, and alignment gaps (green rectangles). Note the conserved absence of stop codons in the +1 frame in the region corresponding to the NS4 ORF. The vertical red line in panel 1 indicates the location of a completely conserved +1 frame AUG codon. One sequence (out of 67) with premature termination codons (PTC) within the NS4 ORF is indicated. Panel 3 shows the probability that the degree of conservation within a given window could be obtained under a null model of neutral evolution of VP6-frame synonymous sites. Panel 4 shows the level of conservation represented by the ratio of the observed number of substitutions within a given window, to the number expected under the null model.

The levels of pairwise nucleotide conservation at synonymous sites within aligned sequences of the VP6 ORF, were used to assess the functional importance of the NS4-ORF. Complete or near-complete VP6-encoding sequences from BTV showed strikingly enhanced conservation in the region corresponding to the NS4 ORF (figure 1), supporting and extending previous computational analyses [17]. Enhanced conservation was also apparent at the 5′ end of the VP6 coding sequence, indicating that this region (like the terminal non-coding regions of orbiviruses) is likely to contain functionally important elements.

Amino acid identity between NS4 of the different *Orbivirus* species compared ranged from 5% to 50%. Highest identity was detected between BTV and EHDV (50%), followed by PHSV and YUOV-1 (30%). Amino acid identity in NS4 between the tick-borne and insect-borne viruses, ranged between 5% to 18% (table 2). Local blast analyses using BLAST-P or TBLAST-N identified significant matches (as defined by the E value in BLAST) between NS4 proteins encoded by other orbiviruses. Analysis of NS4 protein sequences using the pfam program, which uses the hidden Markov model (HMM) based profiles to identify or predict protein functionalities [31], [32], revealed strong similarities to certain conserved functional motifs. AHSV NS4 exhibits strong relatedness over almost its entire length with DUF domains that have helical structures known to be involved in nucleic acid binding and/or modification [33]. Previous analysis of GIV NS4 identified a 72 amino acid fragment (aa 82 to 153) with 39% similarity to dsRNA-binding domains of similar length (approximately 68aa) in other reovirus proteins [ [18] or other dsRNA binding proteins [34]. BTV NS4 (77aa long) also exhibits relatedness (over aa 14–54) to a DUF domain, belonging to the MetJ/Arc repressor superfamily [35], which has a ribbon-ribbon-helix-helix DNA-binding motif, with the beta-ribbon located in and recognising the major groove of operator DNA.

**Table 2:** Percentage amino acid identity values between NS4 of BTV, EHDV, AHSV, GIV, PHSV and YUOV.

	BTV-8(NET2006/04)	EHDV-1(NJ)	AHSV-3	GIV	PHSV
**BTV-8(NET2006/04)**					
**EHDV-1(NJ)**	50				
**AHSV-3**	20	16			
**GIV**	15	18	17		
**PHSV**	15	12	11	10	
**YUOV-1**	9	11	10	5	30

Amino acid identity ranged from 5% to50%. The highest identity exists between BTV and EHDV (50%) followed by PHSV and YUOV-1 (30%). Amino acid identity in NS4 between the tick-borne and insect-borne viruses ranged between 5% and 18%.

BTV NS4 shows strong relatedness to fzo-mitofusin protein, a putative transmembrane GTPase. The fzo protein has a coiled-coil structure and mediates mitochondrial fusion [36]. Another protein family with a coiled-coil structure, which also shows a strong match with BTV NS4, is EMP24_GP25L. Members of this family have been implicated in transporting ‘cargo’ from the endoplasmic reticulum (ER) and are related to the previously described GOLD domain [37], which is always found combined with lipid- or membrane-association domains.

Sequence analyses indicate that PHSV NS4 (111 aa long) contains a coiled-coil domain between aa 75 and 111, YUOV NS4 (113 aa long) contains two coiled-coils domains between aa 5 to 45 and 75 to 105, and AHSV NS4 (143 aa long) contains coiled-coil domains between aa 5 to 85 and aa 110–140. The BTV NS4 (77 aa long) appears to contain only a single coiled coil structure, between aa 27 and 77.

Two overlapping potential nuclear localisation signals’ (NLS) were identified in the aa sequence of PHSV NS4 (positions 86–99: RKLERVEMERKMKK and 95–109: RKMKKSEVNKARRKL) and a single NLS in YUOV (position 99–112: RTPERVESVKKRLN). NLS were also identified in the EHDV NS4 (position 4–13: RHRKGAKRKR) and in BTV NS4 (position 12–24: ‘RKRAAKRLKMQMW). The NLS Mapper predicted 3 potential overlapping NLS in AHSV NS4 (position 4–15: RRTRVKRKRTKY, position 5–15: RTRVKRKRTKY and position 7–16: RVKRKRTKYM). Although all of these NLS were monopartite, the GIV NS4 was found to contain a bipartite NLS (position 113–141: RKRGLEFLLLPLHEYVTHCAKEDIRIYES). The prediction cut-off scores for all these NLS as defined by PredictNSL and cNLS ranged from 4 to 8, indicating dual nuclear/cytoplasmic localisations of a given protein [38].

The aa region 55 to 129 of GIV NS4 showed 29% identity (55% similarity) to aa 1823 to 1890 of UTP20 (a component of the nucleolus).

### Cloning and expression of BTV and GIV NS4

**Figure 2:**
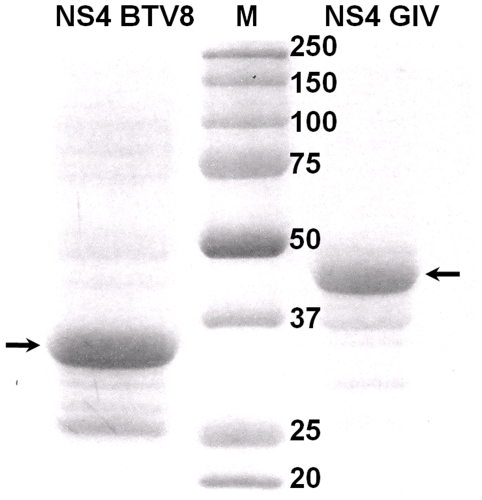
Recombinant NS4 proteins of BTV and GIV. NS4 of BTV and GIV were expressed as soluble GST fusion proteins in C41 bacteria, purified as described in Materials and Methods, then analysed by SDS-PAGE and stained with Coomassie blue. M indicates the molecular weight marker, labelled in kDa.

NS4 of BTV and GIV were successfully cloned into pGEX-4T-2 and expressed in C41 at 28°C, as partially soluble proteins fused to GST (figure 2). The soluble fraction was used in competition assays with DNase I or endoribonucleases belonging to the RNAse III family and in binding assays with dsRNA. In contrast when these proteins were expressed in BL-21 they were totally insoluble and formed inclusion bodies. The inclusion bodies fraction was purified using bugbuster reagent, solubilised and used for immunization of rabbits.

### Western blot analysis of infected cells

**Figure 3:**
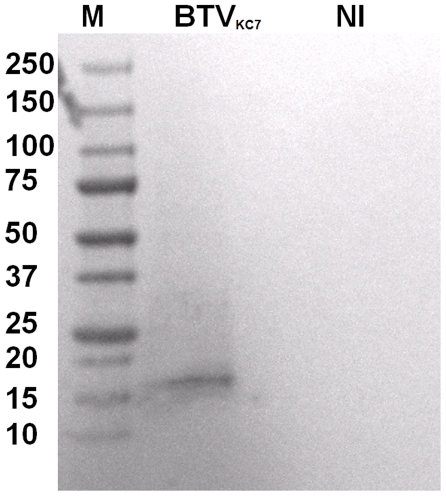
Western blot analysis of BTV-8 in KC cells. BTV-8 infected KC cell lysates analysed by SDS PAGE/Western blot using anti-BTV NS4 antibodies. BTV_KC7_ = KC cells harvested at 7 days post-infection. M indicates the molecular weight marker, labelled in kDa. Lane NI indicates non-infected KC cells which do not show any cross reactivity of anti-BTV NS4 antibody and cellular proteins. NS4 that was identified in infected cells using anti-NS4 antibodies (∼12 kDa) was absent from non-infected cells.

**Figure 4:**
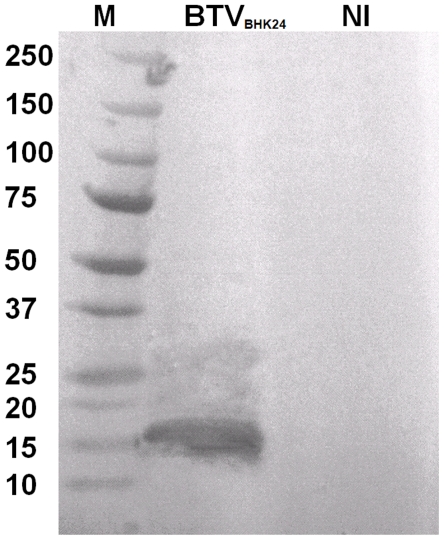
Western blot analysis of BTV-8 in BHK-21 cells. BTV-8 infected BHK-21 cell lysates analysed by SDS PAGE/Western blot using anti-BTV NS4 antibodies. M indicates the molecular weight marker, labelled in kDa. BTV_BHK24_ = BHK-21 cells harvested at 24 hours hours post-infection, respectively. Lane NI indicates non-infected BHK-21 cells which do not show any cross reactivity of anti-BTV NS4 antibody and cellular proteins. NS4 that was identified in infected cells using anti-NS4 antibodies (∼12 kDa) was absent from non-infected cells.

**Figure 5:**
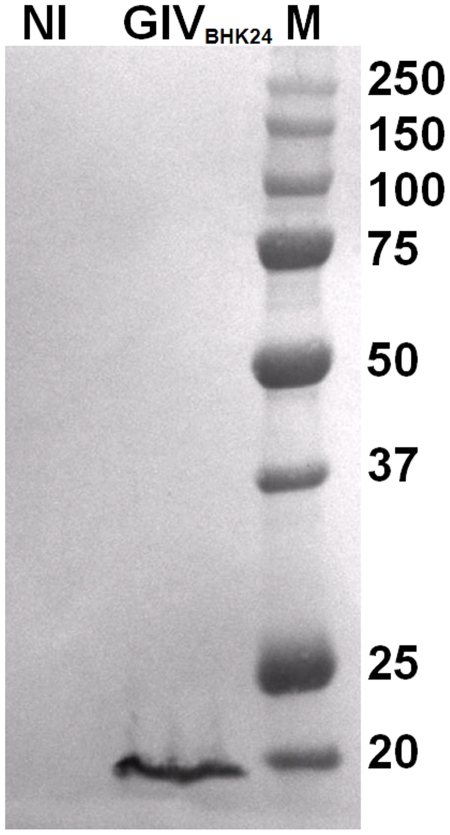
Western blot analysis of GIV in BHK-21 cells. GIV infected BHK-21 cell lysates analysed by SDS PAGE/Western blot using anti-GIV NS4 antibodies. Lane GIV_BHK24_ = BHK-21 cells harvested at 24 hours post-infection. Lane M indicates the molecular weight marker, labelled in kDa. Lane NI indicates non-infected BHK-21 cells which do not show any cross reactivity of anti-GIV NS4 antibody and cellular proteins. A protein was identified by the anti-GIV NS4 antibody in infected cells (approximately 20 kDa) that is absent from non-infected cells.

**Figure 6:**
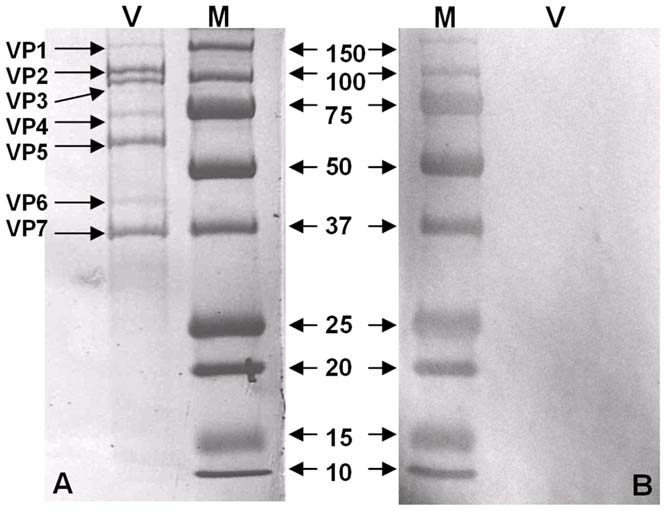
Western blot analysis of purified BTV-8. **A:** SDS-PAGE of purified BTV-8 showing all seven structural proteins stained with Coomassie blue (note the absence of a detectable band of the appropriate size for NS4). **B:** western blot analysis using purified BTV-8 virus particles (as shown in panel D) probed with anti-NS4 antibodies. The reaction is negative, indicating that NS4 is truly non-structural. Lane M: molecular weight markers, labelled in kDa. Lane V: the structural proteins of purified BTV-8 virions are indicated.

**Figure 7:**
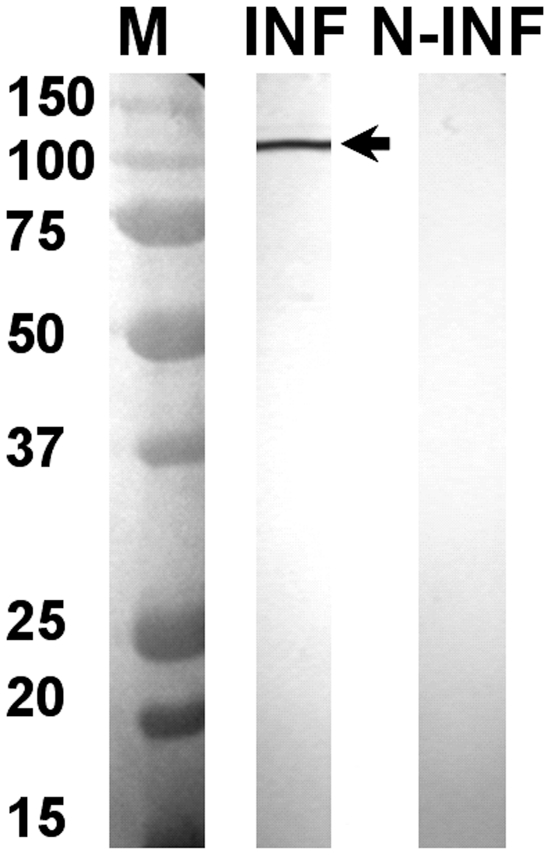
Western blot analysis of BTV-8 infected and non-infected BHK-21 cells using anti-VP2 antibodies. Non-infected (lane N-INF) and BTV-8 infected cells (lane INF) probed with anti-BTV-8 VP2 antibodies raised in mice against recombinant VP2. The antiserum did not cross react with non-infected cells and identified a protein of approximately 110 kDa in infected cells (corresponds to the theoretical size of VP2).

**Figure 8:**
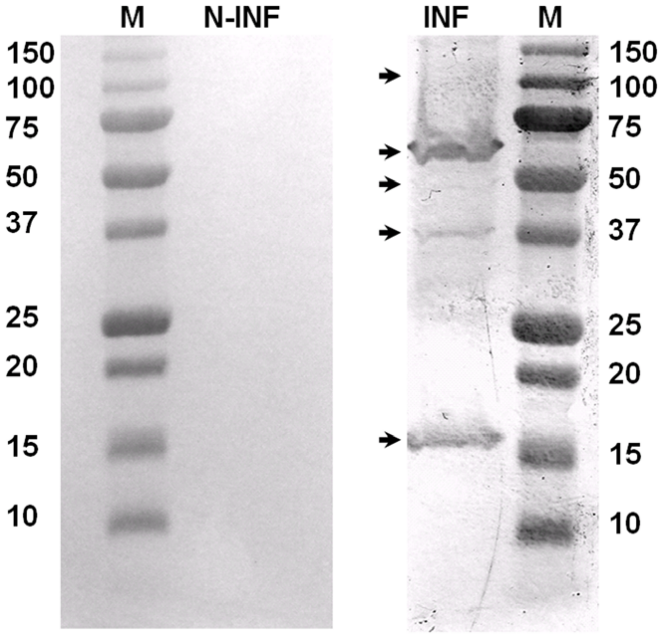
Western blot analysis of BTV-8 infected and non-infected BHK-21 cells using anti-BTV-8 antibodies. Non-infected (lane N-INF) and BTV-8 infected cells (lane INF) probed with anti-BTV-8 immune serum from infected mice. The antiserum did not cross react with non-infected cells and identified several viral proteins in infected cells. Lane M represents the marker labelled in kDa.

Western blots analyses, using rabbit antisera raised against recombinant BTV-8 NS4, showed that NS4 is expressed in BTV-8 infected *Culicoides* KC cells and BHK-21 (figure 3, figure 4). The antiserum identified a single protein band with an apparent molecular weight of approximately 12 kDa in BTV-8 infected cells, which is close to the molecular weight calculated for NS4, from the sequence of Seg-9 (∼10 kDa). The anti-BTV NS4 antiserum is therefore specific to NS4 and does not cross react with other viral proteins. Western blot analysis using non-infected BHK-21 cells, showed that anti-BTV NS4 rabbit antiserum does not cross react with cellular proteins. A similar analysis, using antisera raised against recombinant GIV NS4, identified a protein of approximately 20 kDa in GIV infected cells (figure 5), corresponding to the theoretical molecular weight of NS4 deduced from the sequence of GIV Seg-9. The anti-GIV NS4 antiserum is therefore specific to GIV NS4 and does not cross react with other viral proteins. Western blot analysis using non-infected BHK-21 cells, showed that anti-GIV NS4 rabbit antiserum does not cross react with cellular proteins. Western blot analyses of purified BTV virus particles showed no reaction with anti-BTV NS4 antibodies, indicating that NS4 is ‘non-structural’ (figure 6A, 6B). Figure 7 shows infected and non-infected BHK-21 cells probed with anti-BTV-8 VP2 antibodies raised in mice. Figure 8 shows infected and non-infected BHK-21 cells probed with anti-BTV8 immune serum from experimentally infected mice.

### Identification of NS4 in the nuclear fraction of BTV-8 infected BHK-21 cells

**Figure 9:**
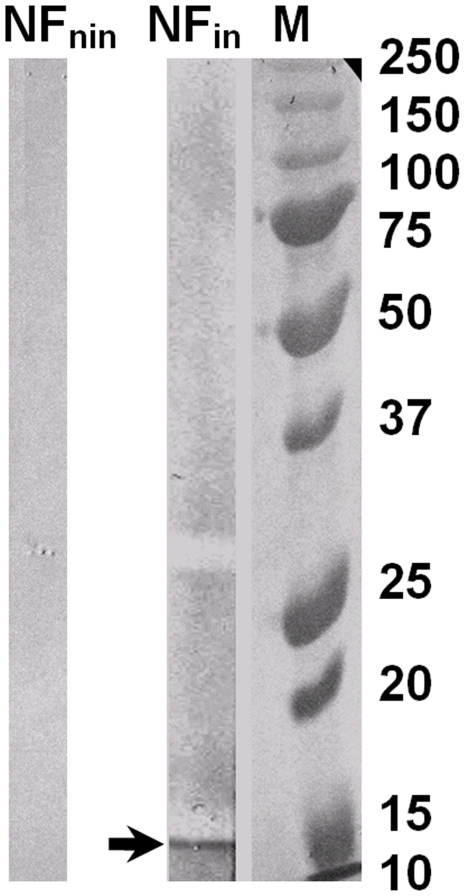
Western blot analysis of the nuclear fraction from BTV-8 infected BHK-21 cells. The nuclear fraction was prepared as described under materials and methods. Infected and non-infected cells were used for the assay. The extracts were analysed by SDS-PAGE/Western blot. Anti-BTV NS4 antibodies identified a protein in the nuclear fraction of infected cells (NF_in_, indicated by an arrow) which is absent from the nuclear fraction of non-infected cells (NF_nin_).

NS4 was identified in the nuclear fraction of BTV-infected BHK-21 cells harvested at 24 hours post-infection by western blot. Rabbit anti-BTV NS4 immune serum, identified the same band in the nuclear extract that was previously identified in infected cell lysates (figure 9). No band was identified in non-infected nuclear extracts.

### Localisation of NS4 in infected cells

**Figure 10:**
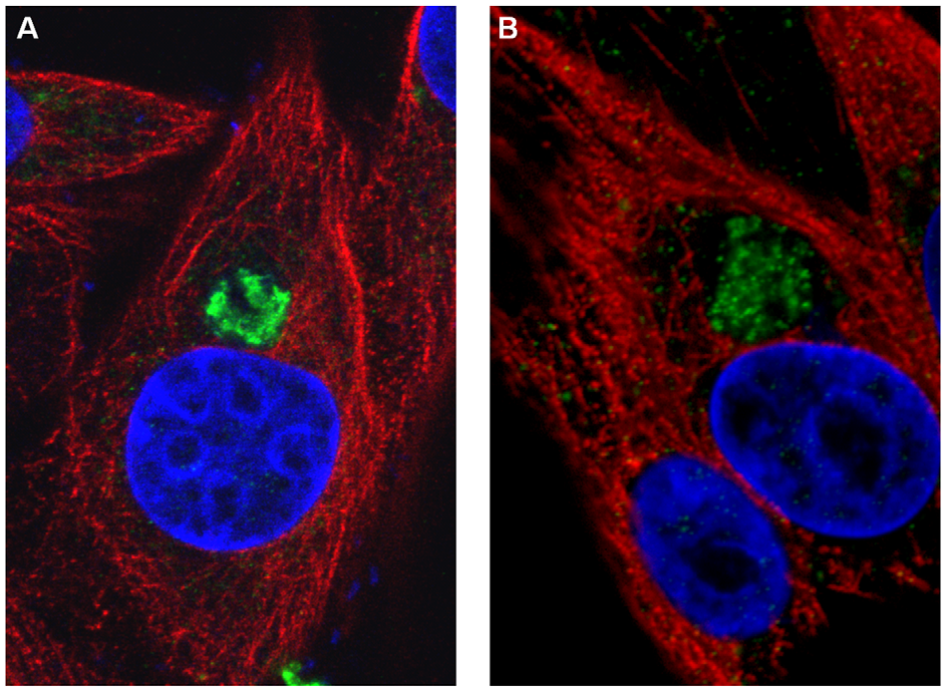
Distribution of NS4 in BTV-8 and GIV infected BHK-21 cells at 4 hours post-infection. **A:** BHK-21 cells infected with BTV-8 showing NS4 mainly in the cytoplasm. **B:** BHK-21 cells infected with GIV showing NS4 both in the cytoplasm. Cells were incubated with anti-BTV-8 NS4, or anti-GIV NS4 rabbit antibodies and anti-alpha tubulin mouse antibodies. Cells were then incubated with Alexa Fluor 488 (green fluorescence) conjugated anti-rabbit IgG and Alexa Fluor 568 (red fluorescence) conjugated anti-mouse.

**Figure 11:**
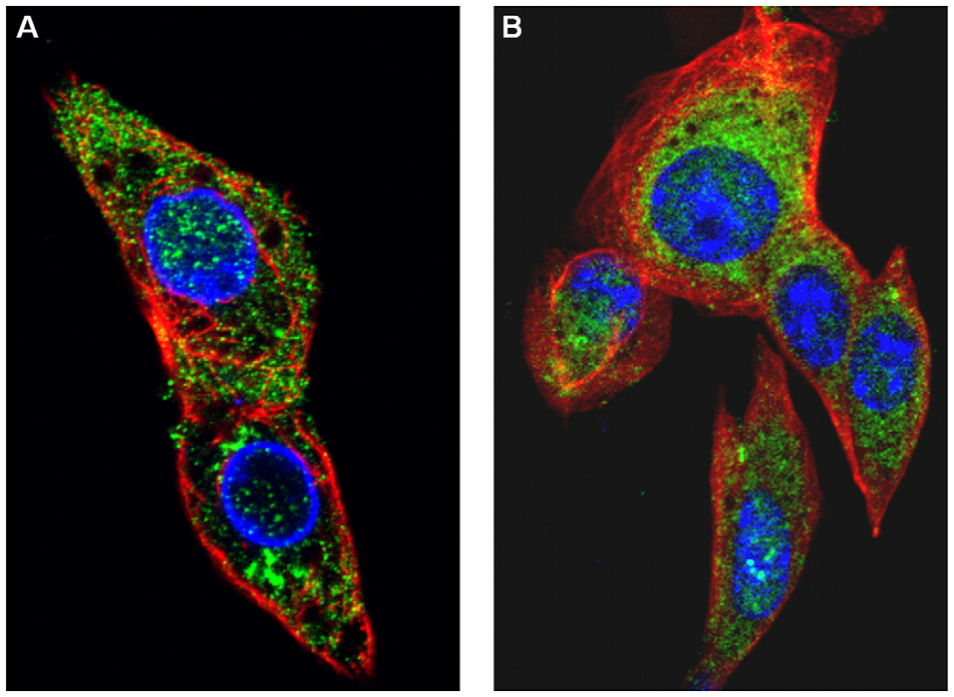
Distribution of NS4 in BTV-8 and GIV infected BHK-21 cells at 24 hours post-infection. **A:** BHK-21 cells infected with BTV-8 showing NS4 both in the cytoplasm and nucleus. **B:** BHK-21 cells infected with GIV showing NS4 both in the cytoplasm and nucleus. Cells were incubated with anti-BTV-8 NS4, or anti-GIV NS4 rabbit antibodies and anti-alpha tubulin mouse antibodies. Cells were then incubated with Alexa Fluor 488 (green fluorescence) conjugated anti-rabbit IgG and Alexa Fluor 568 (red fluorescence) conjugated anti-mouse.

**Figure 12:**
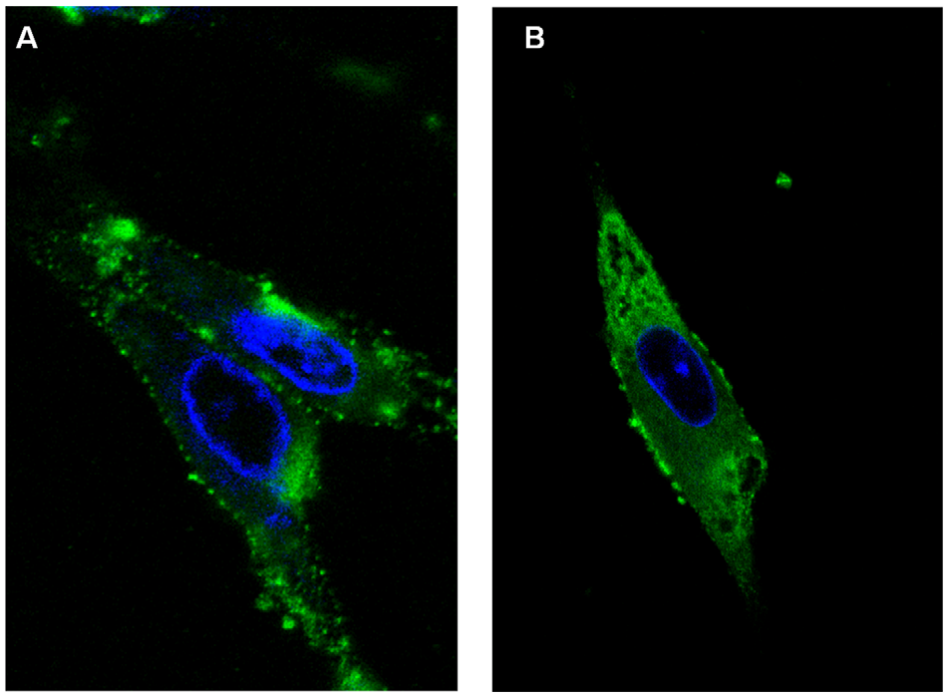
Distribution of NS4 in BTV-8 and GIV infected BHK-21 cells at 72 hours post-infection. **A:** BHK-21 cells infected with GIV showing fluorescence in the cytoplasm and cell membrane but less in the nucleus. **B:** BHK-21 cells infected with BTV-8 showing fluorescence in the cytoplasm and cell membrane but less in the nucleus. Cells were incubated with anti-BTV-8 NS4, or anti-GIV NS4 rabbit antibodies. Cells were then incubated with Alexa Fluor 488 (green fluorescence) conjugated anti-rabbit IgG.

**Figure 13:**
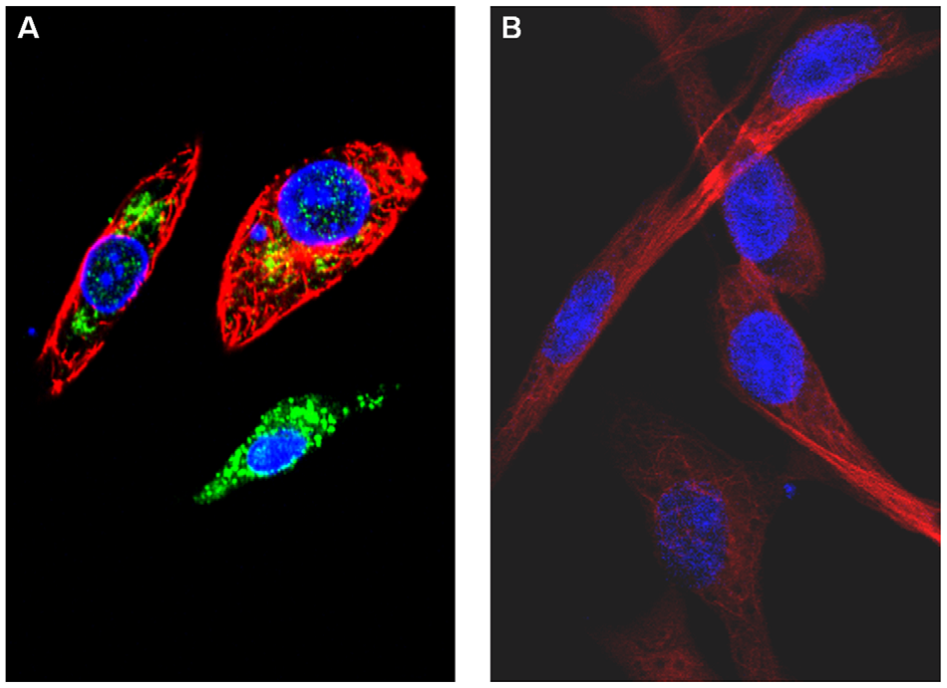
BTV-8 infected and non-infected BHK-21. **A:** BTV-8 infected BHK-21 cells at 36 hours pi, showing cells at different stages of infection. This panel shows a cell (bottom of the panel) with depleted tubulin and an accumulation of the NS4 in the cytoplasm and to a much lesser extent in the nucleus. The panel show cells with a less advanced infection (top) with lower expression of NS4 and an intact alpha-tubulin network. **B:** Non-infected BHK-21 cells stained with DAPI, anti-alpha-tubulin and anti-NS4 antibodies. Cells were incubated with anti-BTV-8 NS4 rabbit antibodies and anti-alpha tubulin mouse antibodies. Cells were then incubated with Alexa Fluor 488 (green fluorescence) conjugated anti-rabbit IgG and Alexa Fluor 568 (red fluorescence) conjugated anti-mouse.

NS4 was detected as early as 4 hours post-infection, mainly in the cytoplasm of BHK-21 cells infected with BTV-8 or GIV (figure 10A, 10B). At 24 hours post-infection, NS4 formed small aggregates throughout the cytoplasm and nucleus, suggesting that it makes specific interactions with itself and/or other infected cell components (figure 11A, 11B). This is consistent with bioinformatic analyses which identified nuclear localisation signals in NS4 of GIV and BTV (as well as YUOV, PHSV, EHDV, AHSV). Although not all cells are morphologically intact at 72 hours post-infection, with GIV or BTV-8, at this stage NS4 was present in the cell membrane (figure 12A, 12B). This is consistent with bioinformatic analysis showing similarities between NS4 and membrane-associated proteins. Another set of cells, which were collected at 36 hours PI contained cells at different stages of infection. Those at an advanced stage of infection had depolymerised and depleted tubulin (figure 13A). No immuno-fluorescence signal was detected when non-infected cells were labelled using anti-NS4 antibodies (figure 13B, indicating that the anti-NS4 antiserum does not cross react with cellular proteins.

**Figure 14:**
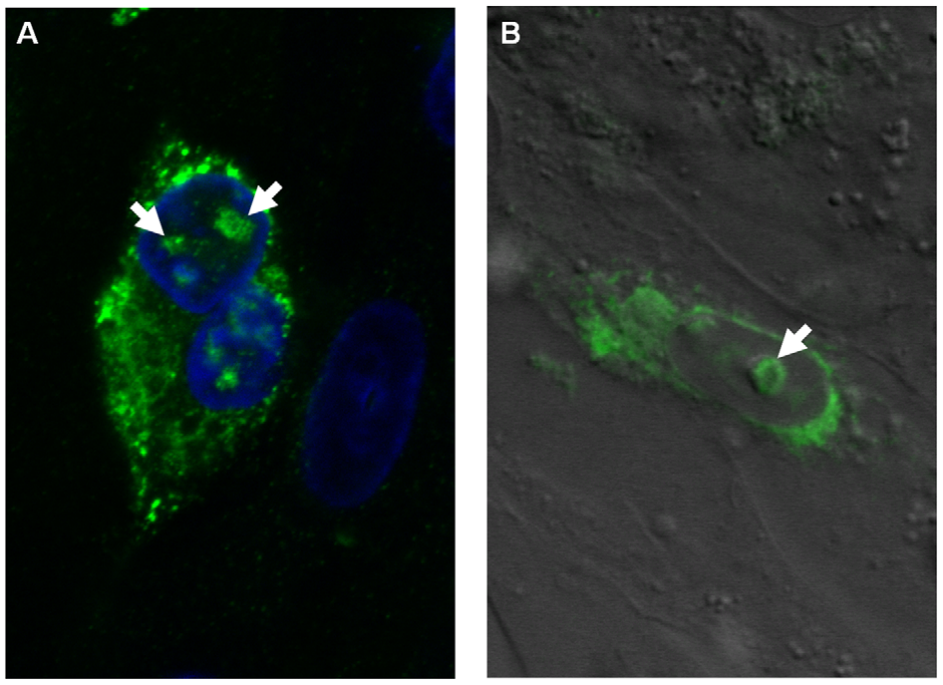
BTV-8 infected BHK-21 showing fluorescence in the nucleoli. **A:** fluorescence signal using anti-NS4 antibodies showing the NS4 in the cytoplasm and nucleus. **B:** Fluorescence signal using anti-NS4 antibodies overlaid onto cells imaged by differential interference contrast showing fluorescence around the nucleus in the cytoplasm and green fluorescence indicated by an arrow overlaid onto the nucleolus. Cells were incubated with anti-BTV-8 NS4 rabbit antibodies and then incubated with Alexa Fluor 488 (green fluorescence) conjugated anti-rabbit IgG.

**Figure 15:**
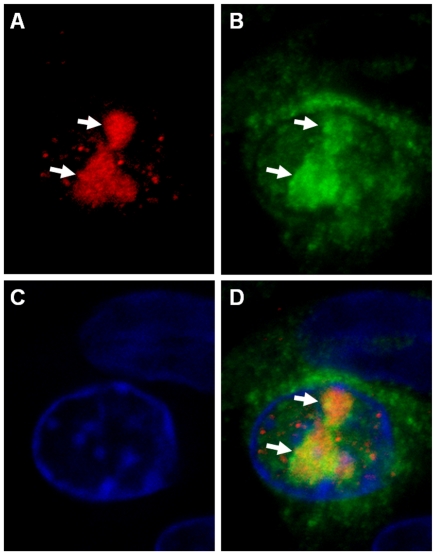
Co-localisation of NS4 and fibrillarin in BTV-8 infected BHK-21. Confocal image of cells infected with BTV-8 showing fibrillarin (**A**: in red) detected by anti-fibrillarin antibody (Serotech), NS4 (**B**: in green) identified by anti-BTV-8 NS4 antibodies, nuclei stained blue with DAPI (**C**) and a merge of these 3 subsets (**D**) showing co-localisation of the BTV-8 NS4 and the fibrillarin (yellow).

Further analyses with confocal microscopy identified nucleolar fluorescence using anti-NS4 antibodies in cells infected with either BTV or GIV. Localisation of the NS4 to the nucleoli was visible by confocal fluorescence as well as by overlaying the fluorescence signal onto cells imaged by differential interference contrast microscopy (figure 14A, 14B). Localisation of NS4 to the nucleoli was confirmed using anti-fibrillarin antibodies, giving a fluorescence signal that was super-imposable on that of NS4 in the nucleoli (figure 15).

### Localization of NS4 in cells transfected with pCI-BTVNS4 or pCI-GIVNS4

**Figure 16:**
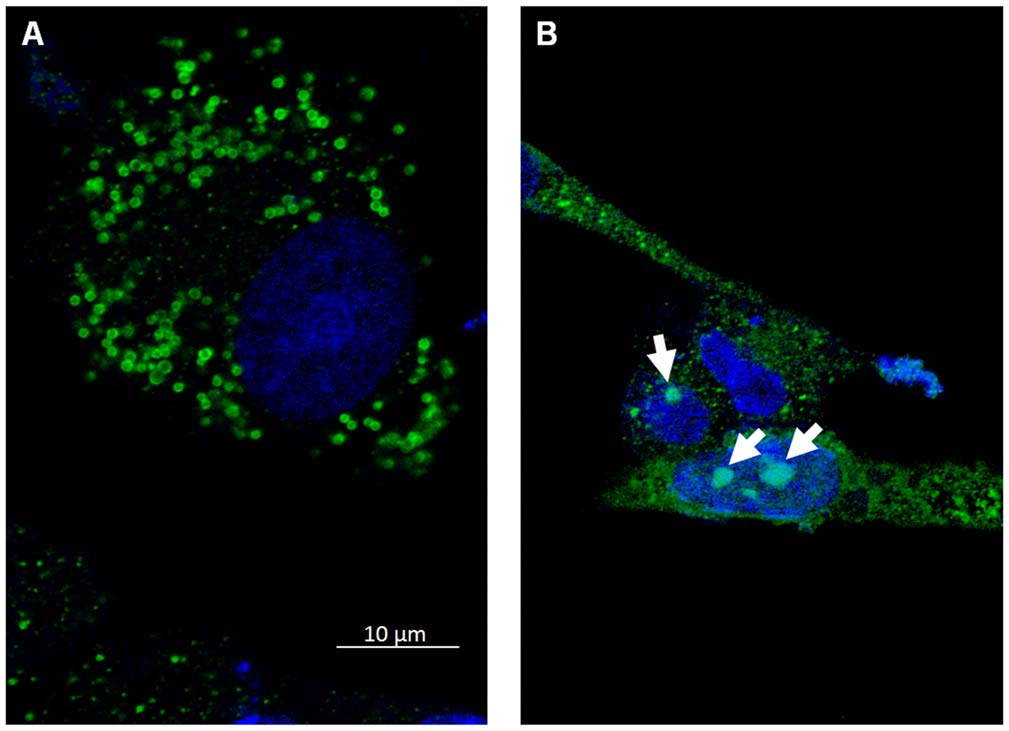
Confocal fluorescence microscopy of BHK-21 cells expressing NS4 of BTV-8. Cells were transfected with pCI-BTVNS4 expressing NS4 of BTV-8. At 48 hours post-transfection, cells were fixed with paraformaldehyde, permeabilised with 0.1% Triton X-100 and incubated with anti-BTV-8 NS4 antibodies. Cells were then incubated with Alexa Fluor 488 (green fluorescence) conjugated anti-rabbit IgG (Invitrogen). **A:** a focal plane of BHK-21 cells expressing NS4 and showing cytoplamsic fluorescence. In a large number of cells, NS4 was found to form spherical bodies (as shown in the figure) having a diameter between 0.7 and 1 µm. **B:** a focal plane of BHK-21 cells showing both cytoplasmic and nucleolar (indicated by arrows) fluorescence. Similar results were obtained with cells transfected with pCI-GIVNS4 expressing NS4 of GIV.

**Figure 17:**
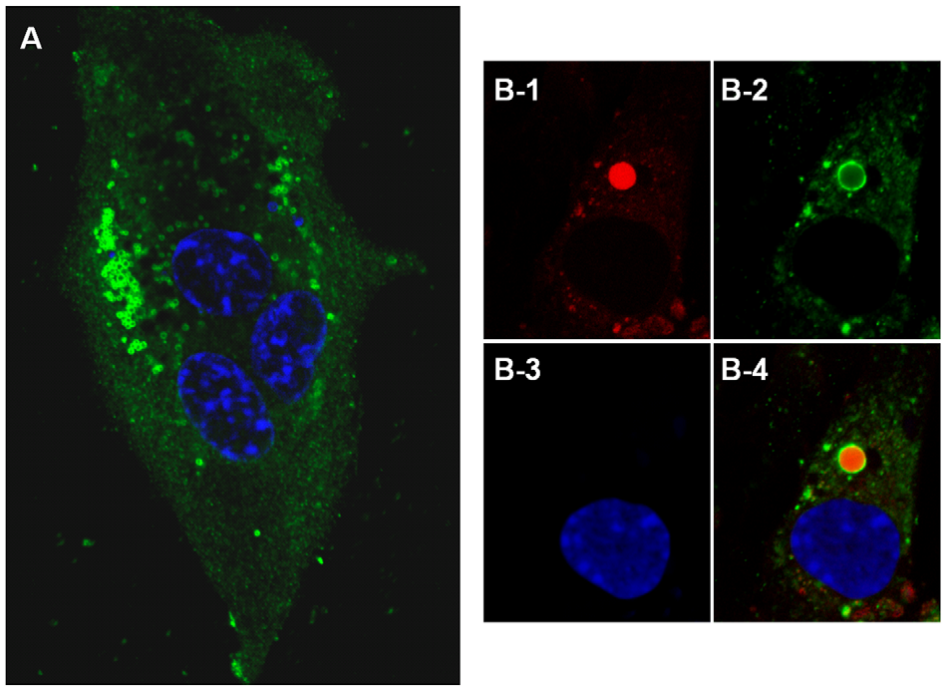
Confocal fluorescence microscopy of GIV or BTV-8 infected BHK-21 cells. **A:** cells were infected with GIV and show spherical bodies (identified by anti-GIV NS4 antibodies) similar to those identified in cells transfected with pCI-BTVNS4. **B:** identification of the spherical bodies (in BTV-8 infected cells) as lipid droplets, by staining with the lipid stain oil-red-O. **B-1:** cells stained with oil-red-O. **B-2:** cells probed with anti-BTV NS4 antibodies. **B-3:** cells stained with DAPI. **B-4:** Co-localisation of NS4 with lipid droplets; oil-red-O stains the lipid droplet in red while the green fluorescence surrounding the lipids indicates BTV NS4.

**Figure 18:**
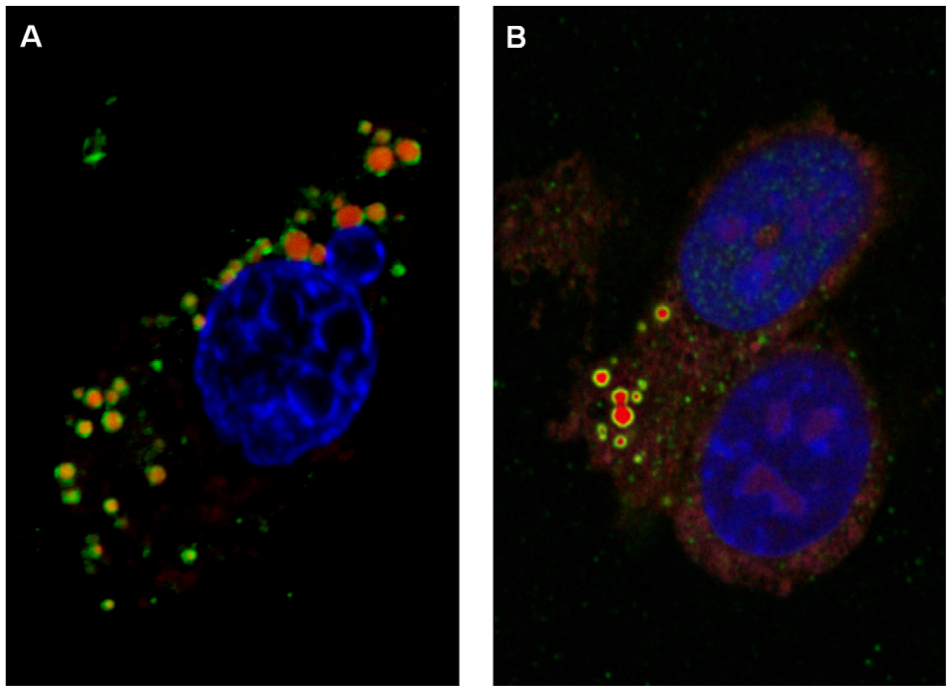
Co-localisation of GIV NS4 or BTV NS4 with lipid droplets in BHK-21 cells. A**:** cells transfected with pCI-GIVNS4 stained with oil-red-O and probed with anti-GIV NS4 antibodies. **F:** Cells infected with BTV-8 stained with oil-red-O and probed with anti-BTV NS4 antibodies.

**Figure 19:**
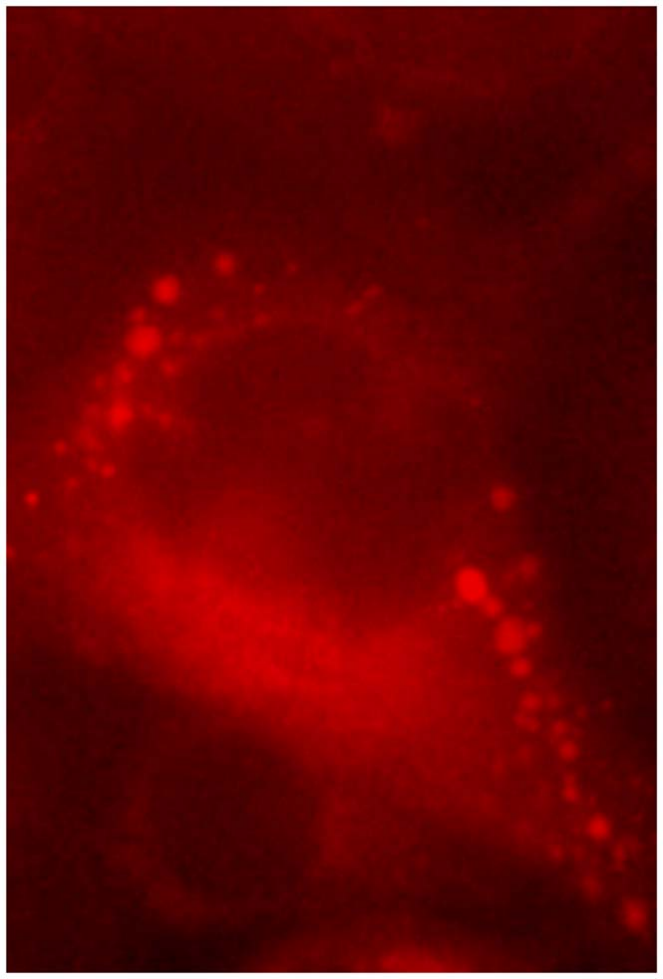
Non-infected BHK-21 cell stained with oil-red-O and anti-BTV NS4.

BHK-21 cells transfected with pCI-BTVNS4 or pCI-GIVNS4 resulted in expression of NS4 in both the cytoplasm and nucleus (figure 16A, 16B). NS4 was also detected in the nucleoli (figure 16B). Expressed NS4 was abundant in the cytoplasm where it formed aggregates similar to those found in infected cells. In many cells NS4 formed spherical bodies with 0.7 and 1 µm in diameter (figure 16A and 16B). Similar spherical bodies were occasionally also observed in cells infected with BTV-8 or GIV (figure 17A). Staining with the lipid stain oil-red-O, showed that these spherical bodies are associations between NS4 and lipid droplets (figure 17B and figure 18A, 18B). These bodies were identified in BTV-8 infected cells (figure 17B and figure 18B), where the oil-red-O stains lipids in the centre of the droplet while NS4 surrounds the lipid droplet. Figure 18A shows cells transfected with pCI-GIVNS4 stained with oil-red-O. Figure 19 shows non-infected cells stained with oil-red-O, where lipid droplets stain with red only. Similar data were recently reported for rotaviruses, where VP2, VP6 or NSP5 were found to associate with lipid droplets [39].

### Western blot analysis of cells transfected with pCI-BTVNS4

**Figure 20:**
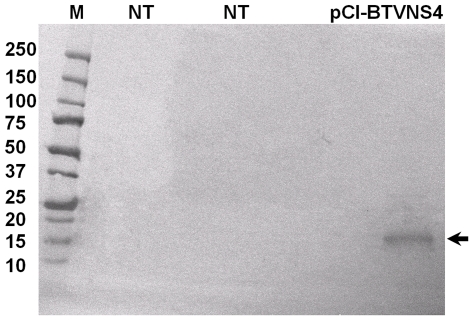
Western blot analysis BHK-21 cells transfected with plasmid pCI-BTVNS4. Cells were transfected with pCI-BTVNS4 expressing NS4 of BTV-8. At 48 hours post-transfection cells were scraped, lysed in sample denaturation buffer and analysed by SDS-PAGE/Western blotting, using BTV-8 immune serum from experimentally infected mice as primary antibody. M indicates the molecular weight marker, labelled in kDa. Lane labelled as pCI-BTVNS4 indicates cells transfected with pCI-BTVNS4 plasmid, NT indicates non-transfected cells. NS4 that was identified in transfected cells using anti-NS4 antibodies (∼12 kDa) was absent from non-transfected cells.

The mice immune serum from an animal infected with BTV-8 identified a protein in cells transfected with pCI-BTVNS4 expressing BTV-8 NS4. The protein band had the same size as that identified by the anti-BTV NS4 rabbit immune serum in BTV-infected cells. No band was identified in non-transfected cells (figure 20).

### Nucleic acid protection assays

**Figure 21:**
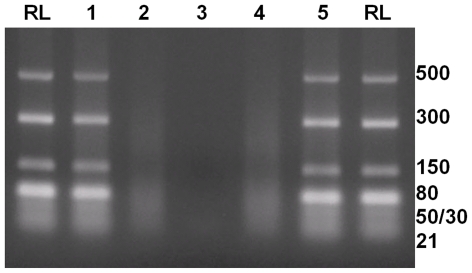
Dicer competition assay. **Lane RL:** dsRNA ladder labelled in base pairs. **Lane 1:** dsRNA ladder pre-incubated with GIV NS4 followed by Dicer. GIV NS4 prevented cleavage by Dicer. **Lane 2:** dsRNA ladder pre-incubated with BTV-8 NS4, followed by Dicer. BTV-8 NS4 did not prevented Dicer from cleaving long dsRNAs into 21 bp long siRNAs. **Lane 3:** ladder incubated with Dicer as a positive digestion-control. **Lane 4**: dsRNA ladder pre-incubated with VP9 of BAV followed by Dicer. VP9 of BAV did not prevent Dicer from cleaving long dsRNAs into 21 bp long siRNA. **Lane 5:** dsRNA ladder incubated with VP9 of BAV. VP9 of BAV did not affect the integrity of dsRNA.

Incubation of a dsRNA ladder (500-21 bp) with Dicer led to cleavage of long dsRNAs, generating 21 bp-long RNAs. Incubation of the dsRNA ladder with BTV NS4 or GIV NS4 alone did not alter dsRNA integrity. dsRNA preincubated with NS4 of GIV was protected against Dicer cleavage, consistent with previous findings regarding the presence of a dsRNA-binding domain. However, BTV NS4 did not protect dsRNA against Dicer and dsRNA was still processed into 21 bp long fragments, as analysed by agarose gel electrophoresis (figure 21). Incubation of dsRNA with BAV outer capsid protein VP9 (as a control) did not affect dsRNA integrity. However, pre-incubation of dsRNA with VP9 then treatment with Dicer resulted in cleavage into 21 bp long fragments (figure 21). Similar results were obtained with RNAse III (data not shown).

**Figure 22:**
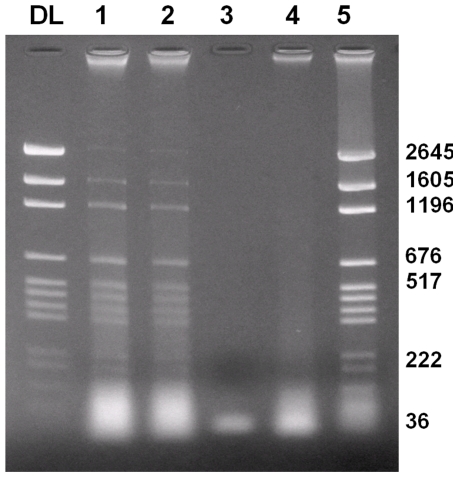
DNase I competition assay. **Lane DL:** dsDNA ladder labelled in base pairs. **Lane 1:** dsDNA ladder pre-incubated with GIV NS4 followed by DNase I. GIV NS4 protected the ladder against DNase cleavage. **Lane 2:** dsDNA ladder pre-incubated with BTV-8 NS4, followed by DNase I, showing that BTV NS4 protected against DNase cleavage. **Lane 3:** Ladder incubated with DNase I as positive control of digestion. **Lane 4**: dsDNA ladder pre-incubated with VP9 of BAV followed by DNAse I. VP9 of BAV did not prevent DNAse I from degrading dsDNA. **Lane 5:** dsDNA ladder incubated with VP9 of BAV. VP9 of BAV did not affect the integrity of dsDNA.

Incubation of a dsDNA ladder (2645–36 bp) with DNAse I led to degradation, while incubation with BTV or GIV NS4 only did not affect dsDNA integrity. However, dsDNA pre-incubated with either BTV or GIV NS4 was at least partially protected against DNAse I (figure 22), as analysed by agarose gel electrophoresis. Incubation of dsDNA with BAV outer-capsid protein VP9 (as a control) did not affect the dsDNA integrity and did not protect the DNA from degradation by DNAse I (figure 22).

### Interaction of NS4 with dsRNA

**Figure 23:**
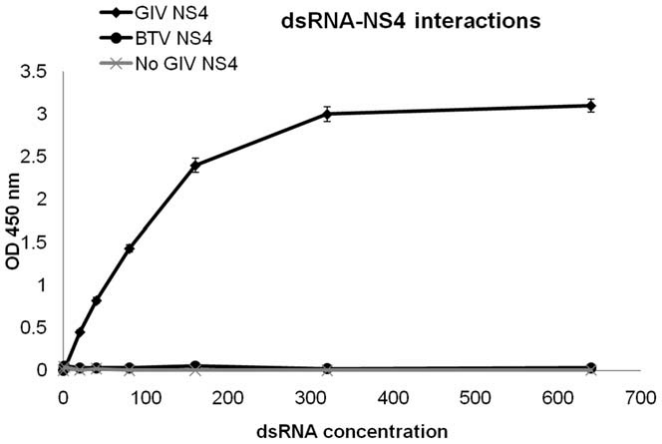
Colorimetric assay to detect interactions of NS4 with dsRNA. The graph shows colorimetric OD readings plotted against concentrations of dsRNA. Increasing concentrations (from 1 to 640 ng) of a biotinylated dsRNA were bound to wells coated with streptavidin. BTV NS4 or GIV NS4 were added to the wells in triplicate. Wells not containing dsRNA/NS4 were included as negative controls. Wells from which dsRNA was omitted, but in which NS4 (BTV or GIV) alone was incubated were also included as controls. Only wells containing the dsRNA to which GIV NS4 was added reacted with anti-GIV antibodies as indicated by increasing OD readings. The readings were almost linear (reaching a plateau at 320 ng of dsRNA) indicating that dsRNA acted as a target for binding of GIV NS4.

In the colorimetric assay to detect NS4-dsRNA binding, the wells devoid of dsRNA were all negative, with a very low background (values close to zero). Negative control wells, containing only dsRNA, also had OD values close to zero (figure 23). Wells containing biotinilated dsRNA and BTV NS4 were also negative, indicating that BTV NS4 does not bind dsRNA. However, the wells containing dsRNA and GIV NS4, had increasing OD values with an almost linear relationship between the fixed NS4 concentration (150 ng/well) and the increasing dsRNA concentration, reaching a plateau at 320 ng of dsRNA/well (figure 23). This confirms the existence of a dsRNA-binding domain in GIV NS4, which is absent from BTV NS4.

## Discussion

Within the family *Reoviridae*, genome segments encoding more than one protein, from distinct, ORFs have been previously reported for the aquareoviruses, fijiviruses, orthoreoviruses, rotaviruses, phytoreoviruses and oryzaviruses [1]. Genome segments of the orthoreoviruses, phytoreoviruses, oryzaviruses and rotaviruses can be bi- or tri-cistronic with overlapping ORFs. Those in the phytoreoviruses (Seg-9 and Seg-12), orthoreoviruses (segment S1) and rotaviruses (Seg-11) were also found to be expressed in infected cell cultures [40], [41], [42], [43], [44]. Translation of overlapping ORFs from reovirus genome segments has usually been shown to be dependent on leaky scanning [41], [43], [44] although scanning-independent ribosome shunting has also been described [42], [45].

An overlapping ORF in Seg-9, designated as ORFx, was recently identified by bioinformatic analysis in both insect-borne and tick-borne orbiviruses [17], [18]. ORFx appeared to encode a protein with the potential to bind dsRNA that was tentatively named as VP6db [18]. However, in line with previous orbivirus protein nomenclature, we have renamed these proteins, based on their theoretical size and absence from the virion, as non-structural protein 4 (NS4). GIV NS4 was previously shown to contain significant aa sequence matches with dsRNA-binding domains [18]. Further analyses of their amino acid sequences indicate that NS4 may be structured as ‘coiled-coils’ and that BTV NS4 exhibits significant relatedness (as identified by the pfam programme) with nucleic acid binding proteins that also have coiled-coils or helical structures and are associated with ER or cell membranes.

**Figure 24:**
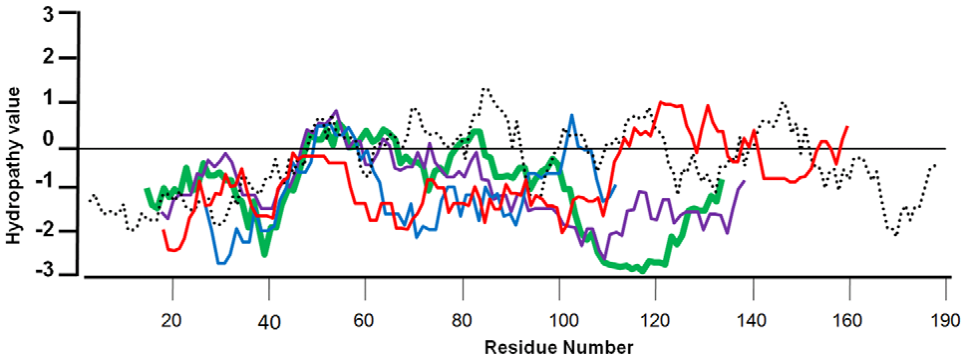
Hydrophobicity profiles of orbivirus NS4. Superimposed hydrophobicity profiles based on a Clustal X generated alignment of orbivirus NS4 amino acid sequences. The residue numbers are relative to NS4 of GIV (the longest NS4 identified to date). GIV NS4 (dashed line), BTV NS4 (blue line), AHSV NS4 (red line), YUOV NS4 (green line) and PHSV NS4 (purple line). The plots show significant similarities (particularly between residues 40–60), with GRAVY values of −1.02 to −1.05, except GIV NS4 which had a GRAVY value of −0.448.

It was suggested that translation of ORFx may be initiated via ‘leaky ribosome-scanning’ [17], although the presence of additional AUG codons between the VP6 initiation codon and the presumed NS4 initiation codon in some orbiviruses (including BTV) suggests that additional mechanisms for bypassing intervening AUG codons may be operating [17]. An A-rich polypurine tract is present upstream of the NS4 ORF in all of the sequenced *Orbivirus* species, except the tick borne St. Croix River virus (SCRV, [46]. The SCRV NS4-ORF (nt 101-379) is interrupted by a stop codon at position 217. Although hydrophobicity profiles of putative NS4 proteins from BTV, AHSV, PHSV, YUOV and GIV as analysed using the Kyte and Doolittle algorithm [22] are somewhat variable, overall they show broadly similar patterns of conserved domains, indicating that the NS4 proteins are generally hydrophilic (figure 24).

BTV-8 infected KC or BHK-21 cells and GIV infected BHK-21 cells all contain NS4, as revealed by western blot analyses and confocal microscopy, confirming the existence of a new and previously un-described protein, encoded by ORFx of orbivirus Seg-9. NS4 has not previously been detected in purified BTV virus or core particles [20]. Western blot analyses of purified BTV-8 particles confirmed that the protein is ‘non-structural’.

Bioinformatic analyses indicate that NS4 contains coiled-coils and is structurally related to other mammalian proteins, with helical or coiled-coil regions. These analyses also suggest that the NS4 may be functionally related to proteins involved in nucleic acid binding, or associated with lipids and membranes. Nuclear localisation signals were predicted in NS4 of PHSV, YUOV, EHDV, BTV, AHSV and GIV. All these proteins are rich in arginine and lysine residues that are essential for NLS [47].

Double-stranded RNA-binding proteins (DRBPs) do not recognize specific nucleotide sequences but interact primarily with A-form double helix RNAs, which differ from the typical dsDNA B-form helix in that the minor groove is shallow and broad while the major groove is narrow and deep. This conformation allows DRBPs to bind non-specifically to dsRNAs. Indeed, the lack of nucleotide binding recognition suggests that target specificity may generally be governed through interactions with other proteins, since many DRBPs bind strongly but non-specifically to any dsRNA structure in vitro. GIV NS4 can protect dsRNA from degradation by RNAse III endoribonucleases, confirming previous sequence analyses indicating the presence of a dsRNA-binding domains [18]. BTV NS4 which is half the theoretical size of its counterpart in GIV, lacks dsRNA binding domains and did not protect dsRNA from Dicer or RNAse III. NS4 of GIV and BTV both failed to protect ssRNA or ssDNA from degradation by RNAse A, or nuclease S1 respectively (data not shown). However, NS4 of both GIV and BTV did protect dsDNA from degradation by DNAse I, indicating an ability to bind dsDNA.

Fluorescent confocal microscopy confirmed that NS4 is expressed in both BTV and GIV infected cells, and starts to accumulate in the cytoplasm and nucleus (as fine aggregates) as early as 4 hours post-infection. However, at 72 hours post-infection NS4 was associated with the cell membrane. This is consistent with analyses suggesting similarities between NS4 and ER- lipid- or membrane-associated proteins [37]. Cells infected with BTV or GIV or transfected with plasmids expressing NS4, showed interaction of NS4 with lipid droplets within the cytoplasm. This is consistent with bioinformatic analysis that identified similarities between NS4 and lipid-associated domains. Similar data were recently reported for VP2, VP6 and NSP5 of rotavirus [39].

Viruses can interact with components of the nucleolus [48], [49] and viral proteins can co-localise with proteins such as nucleolin, B23 and fibrillarin (components of the nucleolus). The use of anti-fibrillarin antibodies identified NS4 in the nucleoli of cells harvested at 24 hours post-infection. Although NS4 was detected in the nucleoli late in infection, anti-fibrillarin antibodies failed to detect fibrillarin. This may reflect BTV induced apoptosis, leading to nuclear condensation and DNA fragmentation, blebbing of the plasma membrane and shrinkage [50], [51], and/or host cell shut-off [4]. Similar findings were reported in rotavirus (another member of the family *Reoviridae*, genus *Rotavirus*) where NSP2 protein was found to cause depolymerisation of tubulin [52].

As part of their replication strategy, viruses can use nucleolar components to favour viral transcription and translation, or alter the cell cycle [48], [49]. Western blot analysis indentified NS4 in the nuclear fraction of BTV infected cells, while immunofluorescence confocal microscopy co-localised NS4 to the nucleolus. GIV-NS4 showed sequence similarity to UTP20, a small subunit processome component and a component of the nucleolus. UTP20 is part of the U3 small nucleolar RNA (snoRNA) protein complex (U3 snoRNP) and is involved in 18S rRNA processing [53]. Whether NS4 interferes with the processing of the 18s rRNA remains to be clarified in future work.

The ability of GIV NS4 to protect dsRNA from cleavage by endoribonucleases of the RNAse III family and its ability to bind dsRNA agree with sequence analyses that indicated the presence of a dsRNA binding domain in GIV NS4 [18]. The inability of BTV NS4 to protect dsRNA from cleavage by endoribonucleases of the RNAse III family and its inability to bind dsRNA in a plate-based colorimetric assay are in agreement with sequence analyses that failed to detect a dsRNA binding domain in its aa sequence [18]. Other reoviruses dsRNA-binding proteins include Sigma 3 of mammalian orthoreovirus (found in both the cytoplasm and nucleus), and pns10 of rice dwarf virus [54], [55].

SCRV, which persistently infects tick cells but does not grow in mammalian cells, appears to have a non-functional NS4 ORF that is interrupted by a stop codon. These observations suggest that NS4 expression could play a role in productive infection of mammalian cells.

The data presented here show that the orbivirus genome encodes four distinct non-structural proteins (NS1-NS4). NS1 and NS3 play an important role in orbivirus exit mechanisms from infected cells [15]. BTV infects mammalian cells, usually resulting in a lytic infection, while infection of KC cells derived from the BTV vector *Culicoides sonorensis*, become persistently infected with little or no evidence of cell lysis [56], [57]. Previous work showed that intracellular expression of an NS1 specific antibody fragment (scFv) destabilised the formation of NS1 tubules in BTV infected cells [14]. As a consequence, cells became persistently infected and viruses exited by budding instead of via cell lysis. Although BTV NS3 is effectively expressed in insect cells [15], it is much less abundant in mammalian cells [4]. It was suggested previously [14] that the relative levels of NS1 to NS3 synthesised during infection dictate the fate of cellular pathogenesis as of whether the virus exit occurs by lysis or budding.

The rapid accumulation of NS4 in the cytoplasm as early as 4 hours post-infection suggests that this protein plays an early role in the virus replication cycle. At 72 hours post-infection NS4 was absent from the nucleus which could be the consequence of changes affecting the nucleus and the integrity of the nuclear membrane. The presence of the NS4 in the plasma membrane late in infection suggests that it may play a role, alongside NS1 and NS3, in virus exit. Further co-localisation studies will be carried out to assess NS4 interactions with other viral or cellular protein components.
